# Redundancy and Molecular Evolution: The Rapid Induction of Bone Formation by the Mammalian Transforming Growth Factor-β_3_ Isoform

**DOI:** 10.3389/fphys.2016.00396

**Published:** 2016-09-08

**Authors:** Ugo Ripamonti, Raquel Duarte, Ruqayya Parak, Caroline Dickens, Therese Dix-Peek, Roland M. Klar

**Affiliations:** ^1^Bone Research Laboratory, Faculty of Health Sciences, School of Oral Health Sciences, University of the WitwatersrandJohannesburg, South Africa; ^2^Department of Internal Medicine, Faculty of Health Sciences, School of Clinical Medicine, University of the WitwatersrandJohannesburg, South Africa; ^3^Department of Oral Biological Sciences, School of Oral Health Sciences, University of the WitwatersrandJohannesburg, South Africa

**Keywords:** induction of bone formation, transforming growth factor β_3_, redundancy, primates, molecular evolution

## Abstract

The soluble osteogenic molecular signals of the transforming growth factor-β (TGF-β) supergene family are the molecular bases of the induction of bone formation and postnatal bone tissue morphogenesis with translation into clinical contexts. The mammalian TGF-β_3_ isoform, a pleiotropic member of the family, controls a vast array of biological processes including the induction of bone formation. Recombinant hTGF-β_3_ induces substantial bone formation when implanted with either collagenous bone matrices or coral-derived macroporous bioreactors in the *rectus abdominis* muscle of the non-human primate *Papio ursinus*. In marked contrast, the three mammalian TGF-βs do not initiate the induction of bone formation in rodents and lagomorphs. The induction of bone by hTGF-β_3_/preloaded bioreactors is orchestrated by inducing fibrin-fibronectin rings that structurally organize tissue patterning and morphogenesis within the macroporous spaces. Induced advancing extracellular matrix rings provide the structural anchorage for hyper chromatic cells, interpreted as differentiating osteoblasts re-programmed by hTGF-β_3_ from invading myoblastic and/or pericytic differentiated cells. *Runx2* and *Osteocalcin* expression are significantly up-regulated correlating to multiple invading cells differentiating into the osteoblastic phenotype. Bioreactors pre-loaded with recombinant human Noggin (hNoggin), a BMPs antagonist, show down-regulation of *BMP-2* and other profiled osteogenic proteins' genes resulting in minimal bone formation. Coral-derived macroporous constructs preloaded with binary applications of hTGF-β_3_ and hNoggin also show down-regulation of *BMP-2* with the induction of limited bone formation. The induction of bone formation by hTGF-β_3_ is *via* the BMPs pathway and it is thus blocked by hNoggin. Our systematic studies in *P. ursinus* with translational hTGF-β_3_ in large cranio-mandibulo-facial defects in humans are now requesting the re-evaluation of “*Bone: formation by autoinduction*” in primate models including humans.

## Redundancy and the induction of bone formaton by multiple homologous yet different soluble osteogenic molecular signals of the transforming growth factor-β supergene family

The osteogenic activity by a series of extracellular matrices, including the renal parenchyma, was discovered after ligature of the vascular peduncle in lagomorphs (Sacerdotti and Frattin, 1901: reviewed by Ripamonti et al., 2006; Ripamonti, 2010). Subsequently, several research laboratories attempted to isolate and purify the elusive “*osteogenic activity*” present in several extracellular matrices, including uroepithelium, bone and dentine matrices, that was postulated by the classic studies of Sacerdotti and Frattin (1901), Levander (1945), Moss (1958), Huggins (1968), Lacroix (1945), Friedenstein (1962), Trueta (1963), Urist (1965), Sampath and Reddi (1981) and Sampath et al. (1987). Urist (1965), in his classic contribution to Science, conclusively showed the bone induction activity of demineralized bone matrix in rodents and even reported the implantation of demineralized bone matrix in mandibular defects of human patients (Urist, 1965).

The definition of the “*bone induction principle*” and of the morphogenetic capacity of intact demineralized bone matrices (Urist, 1965; Urist et al., 1967, 1968; Reddi and Huggins, 1972) later yielded the isolation of soluble and insoluble signals by Reddi and co-authors (Sampath and Reddi, 1981). These classic experiments reported the chaotropic extraction of the intact demineralized bone matrix into a soluble signal (the protein extract), and an insoluble signal or residue, the inactive insoluble collagenous bone matrix (Sampath and Reddi, 1981, 1983; Reddi, 2000). Implantation of the lyophilized soluble signal or of the insoluble signal or substratum did not result in the induction of bone formation, indicating that the chaotropic dissociative extraction of the bone matrix disrupted the bone induction cascade of the intact demineralized bone matrix (Sampath and Reddi, 1981, 1983; Reddi, 1997).

The reconstitution of the soluble with the insoluble signal or substratum restored the biological activity of the intact demineralized bone matrix (Sampath and Reddi, 1981). This operational reconstitution of a soluble signal with an insoluble signal or substratum paved the way for the chromatographic purification of the soluble signals extracted from the intact demineralized bone matrices (Sampath and Reddi, 1981, 1983; Sampath et al., 1987; Reddi, 2000). The resolution of the biological problem of the “*bone matrix in the solid state*” (Reddi, 1997) facilitated the isolation, purification and molecular cloning of a novel family of protein initiators, collectively called the bone morphogenetic proteins (BMPs) (Reddi, 2000; Ripamonti, 2006). The BMPs are endowed with the property of initiating *de novo* induction of endochondral bone formation when implanted in heterotopic extraskeletal sites of a variety of animal models including primates (Reddi, 2000; Ripamonti, 2006; Ripamonti et al., 2006).

The BMPs subfamily belongs to the transforming growth factor-β (TGF-β) supergene family (Wozney et al., 1988; Kingsley, 1994; Reddi, 2000; Ripamonti, 2006). Approximately 60 members of the TGF-β superfamily have been identified and can be placed into one of two main subfamilies. Both subfamilies have a central signaling pathway operating downstream of ligand binding (Shi and Massagu, 2003). The evolutionary importance of the TGF-β superfamily is emphasized by the conserved characteristics of BMP/TGF-β signaling and points to the vital role that these factors play in vertebrate physiology (Schmierer and Hill, 2007).

Conserved canonical BMP/TGF-β signaling is comprised of the BMP/TGF-β ligands which bind cell surface receptors to relay the signal via the transducers, receptor regulated -Smads (R-Smads) (Feng and Derynck, 2005). The activated Smads in turn interact with downstream effector molecules, such as Runx2 to effect bone differentiation. Non-canonical signaling (p38 mitogen-activated protein kinase, Smad independent) also activates Runx2 to activate mesenchymal stem cell differentiation. The synchronized activity of canonical and non-canonical signaling is crucial for the formation of bone. An important feature of BMP/TGF-β regulation in osteogenesis is the interaction of components of the BMP/TGF-β signaling pathways with other pathways (Chen et al., 2012). This signaling cross-talk is responsible for imparting the diversity, flexibility and intricacies of the BMP/TGF-β pleiotropic functions. The interaction of TGF-β/BMP signaling with other major pathways, most notably the Wnt pathway (Issack et al., 2008; Kim et al., 2013), have been studied in detail. The highly conserved Runx2 transcription factor plays a key role in integrating the signals from the collaborating pathways (reviewed in Rahman et al., 2015).

Morphogens of the TGF-β superfamily play pleiotropic roles in axial patterning, tissue morphogenesis and organogenesis in both vertebrates and invertebrates (Wozney et al., 1988; Reddi, 2000; Ripamonti et al., 2005; Ripamonti, 2006). The realization of the pleiotropic activity of the soluble signals of the TGF-β supergene family has dramatically advanced our molecular and cellular understandings of tissue induction and morphogenesis (Reddi, 2000; Ripamonti et al., 2004; Ripamonti, 2006). The elusive putative osteogenic proteins were finally isolated and purified to homogeneity from the extracellular matrix of bone (Ripamonti, 2006). The isolated and later recombinant molecular signals acted as morphogens, first defined by Turing as “form generating substances” (Turing, 1952). Morphogens of the TGF-β supergene family initiate the induction of bone formation as a recapitulation of embryonic development (Reddi, 2000; Ripamonti et al., 2000b, 2004; Ripamonti, 2006). Any perturbations to TGF-β/BMP regulation results in the pathogenesis of many diseases including those of the human skeleton, such as fibrodysplasia ossificans progressiva (FOP), a disabling disease due to mutations of the conserved TGF-β type I receptor (Kaplan et al., 2009).

Experiments by Sampath et al. (1993) have shown that the prerogative of the induction of bone formation, so far solely ascribed to the BMPs family of proteins, could be extended to additional members of the TGF-β supergene family. They reported endochondral osteoinductive activity of human recombinant decapentaplegic (dpp) and 60A gene products of the fruit fly *Drosophila melanogaster*, in the rodent subcutaneous bioassay (Sampath et al., 1993).

The TGF-β family comprises three mammalian isoforms, the TGF-β_1_, -β_2_, and -β_3_ proteins. In *Homo sapiens* each of the isoforms are encoded by different genes located at different regions of the genome (Fujii et al., 1986; Barton et al., 1988; ten Dijke et al., 1988). They are highly homologous with TGF-β_1_ and TGF-β_2_ exhibiting 71.4% amino acid homology and TGF-β_3_ sharing 76% and 80% sequence similarity with TGF-β_1_ and TGF-β_2_, respectively (Marquardt et al., 1987; Yue and Mulder, 2001). The TGF-β superfamily is considered an ancient protein family arising approximately 800 million years ago coinciding with the origin of metazoans. Various components of the signaling pathways have been detected in the most ancient of the metazoa, Cnidaria, and sponges (Blitz and Cho, 2009).

Members of the TGF-β superfamily are deeply conserved and share a high degree of homology, especially in the C-terminus region (Burt, 1992; Konikoff et al., 2010). The high levels of amino acid similarity in the ligands, receptors and target Smads point to a co-evolution predating the common ancestor of *C. elegans, D. melanogaster* and *Mus musculus* (Konikoff et al., 2010). Members of the TGF-β family share a number of common structural features. They all have an N-terminal signal sequence that is removed before the protein is secreted; they have a large pro-protein region that is also removed prior to secretion but that assists in the formation of the dimer for the C-terminal biologically active ligand and they have a ligand domain approximately 110 amino acids in length that contains a standard pattern of 6 cysteines (Kahlem and Newfeld, 2009). Many family members have an additional, seventh, cysteine residue that is centrally located and involved in the formation of the ligand dimer.

**Figure 1:**
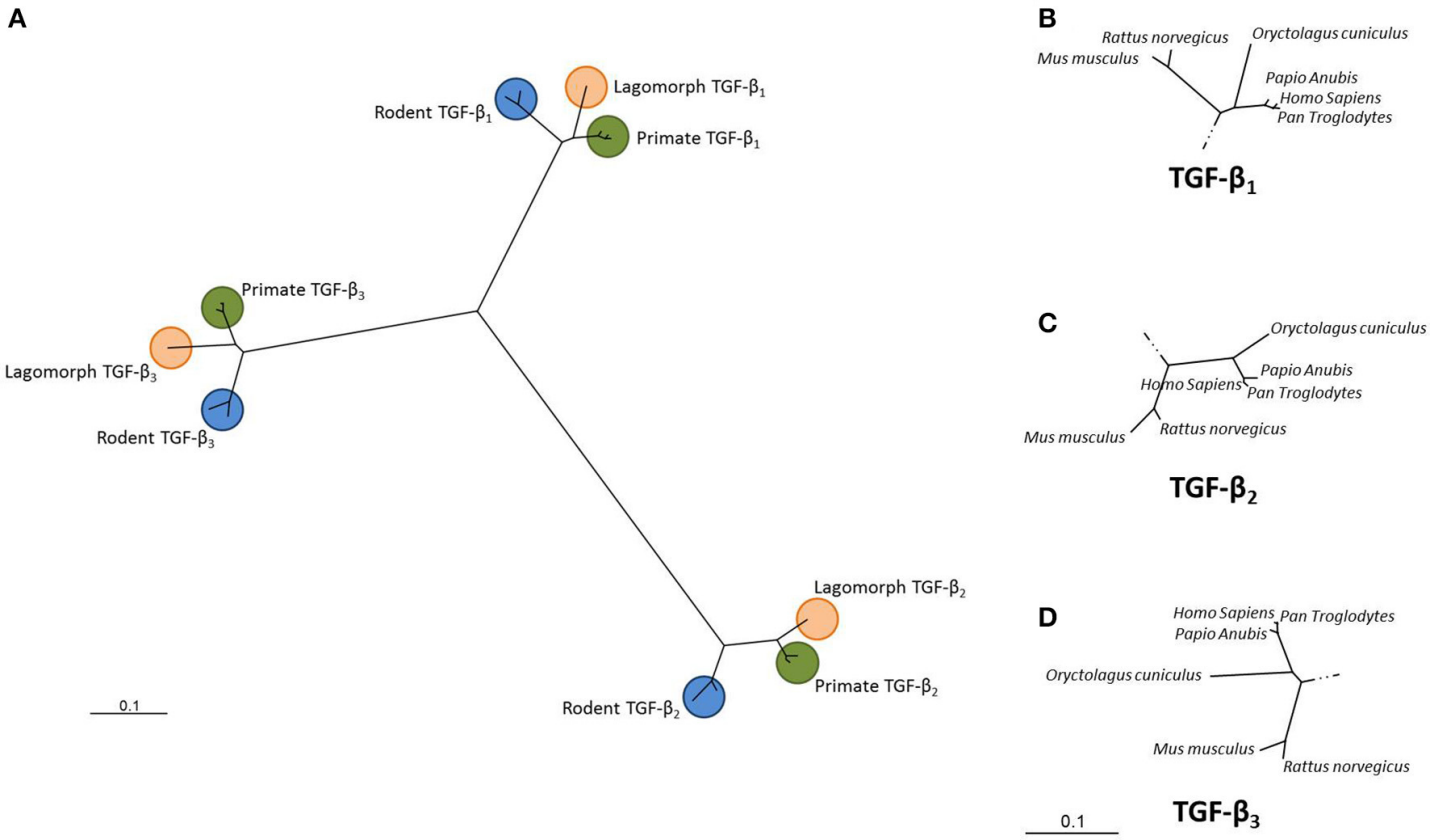
**Unrooted dendrogram showing the relationship between the TGF-β_**1**_, TGF-β_**2**_ and TGF-β_**3**_ isoforms**. Sequences from *Homo sapiens, Pan troglodytes, Papio anubis, Oryctolagus cuniculus, Mus musculus* and *Rattus norvegicus* were downloaded from GenBank for each of the three TGF-β isoforms. Sequences were aligned using Clustal X and a phylogenetic tree constructed using the Neighbor-joining algorithm (PHYLogeny Inference Package, version 3.6; Felsestein, 1995). **(A)** Overall relationship while **(B–D)** show each clade in more detail. (GenBank accession numbers: NM_000660; NM_001135599; NM_003239; XM_009435655; XM_001172158; XM_001161669; XM_009194559; XM_003893100; XM_003902079; XM_008249704; NM_001082660; XM_002719635; NM_011577; NM_009367; NM_009368; NM_021578; NM_031131; NM_013174).

Phylogenetic analysis relates the distances between branch points in a phylogenetic tree to evolutionary distance (Burt, 1992). Using phylogenetics, two large subfamilies have been identified within the TGF-β superfamily, the Decapentaplegic/bone morphogenetic protein (Dpp/BMP) subfamily and the TGF-β/Activin subfamily (Kahlem and Newfeld, 2009). Dpp from *D. melanogaster* and BMP-2 and BMP-4 from *M. musculus* share 75% homology. In fruit flies, human BMP-2 and BMP-4 have been shown to rescue *dpp* mutant phenotypes. Figure 1 shows a phylogenetic tree depicting the evolutionary relatedness of the TGF-β isoforms in the primates, rodents and lagomorphs. The high degree of conservation within these isoforms is clearly demonstrated and the tight clustering within the primates shows a high degree of conservancy between these sequences.

The extension of the unique prerogative of the induction of bone formation from BMPs to other molecularly and functionally unrelated members of the TGF-β supergene family has indicated the apparent redundancy of gene and gene products initiating the induction of bone formation in non-human primates (Ripamonti et al., 1997, 2000a, 2004, 2008a, 2010; Ripamonti, 2006; Ripamonti and Roden, 2010), and by extension, to human primates (Ripamonti and Ferretti, 2016). Our laboratories have shown the rapid and substantial induction of bone formation in full thickness mandibular defects prepared in *Papio ursinus* (Ripamonti and Ferretti, 2016). Translational research in clinical contexts from *P. ursinus* to severe massive mandibular discontinuities in selected human patients culminated in the regeneration of the avulsed body and *ramus* of the newly formed mandible, with restoration of the avulsed coronoid process (Ripamonti and Ferretti, 2016).

Of interest, since our first paper reporting the endochondral osteoinductivity of the hTGF-β_1_ isoform (Ripamonti et al., 1997), no other research laboratories have ever reported any study on the bone inductive activity of the mammalian TGF-β isoforms, and the reported human experimentation on bone tissue engineering by the recombinant hTGF-β_3_ isoform is so far the only reported data world-wide (Ripamonti, 2016; Ripamonti and Ferretti, 2016). There are thus no other studies to compare with our own published work and our continuous experimentation to correlate the induction of bone formation by the hTGF-β_3_ isoform from the laboratory benches, to *P. ursinus* and to *H. sapiens* in clinical contexts (Ripamonti, 2016).

**Figure 2:**
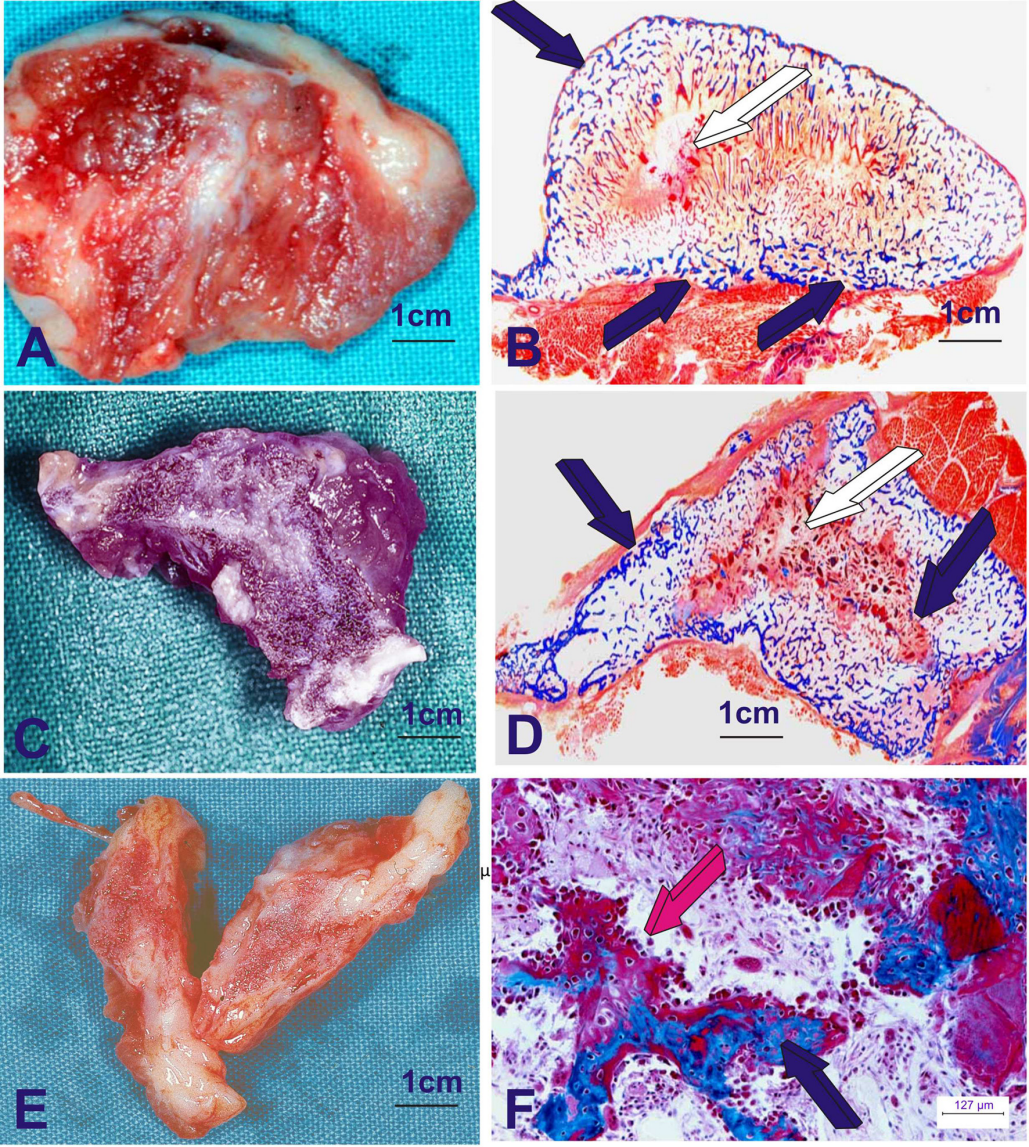
**Heterotopic induction of bone formation in the ***rectus abdominis*** muscle of adult Chacma baboon ***Papio ursinus*** by 125 μg of recombinant human transforming growth factor-β_**3**_ (hTGF-β_**3**_)**. hTGF-β_3_ was reconstituted with allogeneic insoluble collagenous bone matrix as carrier. The operational reconstitution of the soluble molecular signal, the hTGF-β_3_, with the collagenous matrix, the insoluble signal, sets into motion the striking osteogenic activity of the hTGF-β_3_ isoform, inducing rapid and substantial bone formation. **(A,C,E)** Series of large corticalized newly formed ossicles 7–9 cm in length in the *rectus abdominis* muscle harvested on day 30 generated *de novo* after intramuscular implantation of 125 μg hTGF-β_3_. **(B,D,F)** Undecalcified histological sections cut at 6 μm of the newly formed large corticalized ossicles after implantation in the *rectus abdominis* muscle of *Papio ursinus* and harvested on day 30 (Ripamonti et al., 2008a). **(B,D)** Whole mounted undecalcified sections highlighting induction of large corticalized mineralized (dark blue arrows) ossicles harvested on day 30. **(F)** High power view detailing the rapid induction of bone formation by a plurality of contiguous plumped osteoblastic cells secreting osteoid matrix (magenta arrow) surfacing newly formed mineralized bone (dark blue arrow). 6 μm undecalcified sections, stained free-floating with modified Goldner's trichrome stain.

Experimentation reporting the induction of bone formation by the hTGF-β_3_ isoform has shown that the human recombinant protein is the most powerful inductive morphogen so far tested in primates (Figure 2; Ripamonti et al., 2008a,b, 2010, 2014, 2015). The Chacma baboon *P. ursinus* displayed unique results when compared to rodents, lagomorphs and canines. The question that thus arose was: why in primates, and in primates only, are there several related homologous yet molecularly different morphogens that set into motion the induction of bone formation? (Figure 2). Until recently, this question still remained unanswered and needed to be assigned (Klar et al., 2014; Ripamonti et al., 2014, 2015).

The presence of multiple molecular forms with osteogenic activity in heterotopic intramuscular sites of primates poses a major therapeutic challenge in terms of single recombinant osteogenic protein selection (Ripamonti and Reddi, 1994; Ripamonti et al., 2004, 2007). The mosaicism of expression of different BMPs during skeletogenesis and pattern formation has indicated that different regions of the skeleton including the craniofacial skeleton may have different *ratios* of isoforms within the skeleton, reflecting a therapeutic significance (Ripamonti and Duneas, 1998; Ripamonti, 2006). The mosaicism of expression may indicate a site-specific regulatory role of different BMPs *in vivo* and underscores the therapeutic importance of site targeting with exogenous single or binary applications of specific osteogenic gene products of the TGF-β supergene family (Ripamonti and Duneas, 1998; Ripamonti, 2006).

**Figure 3:**
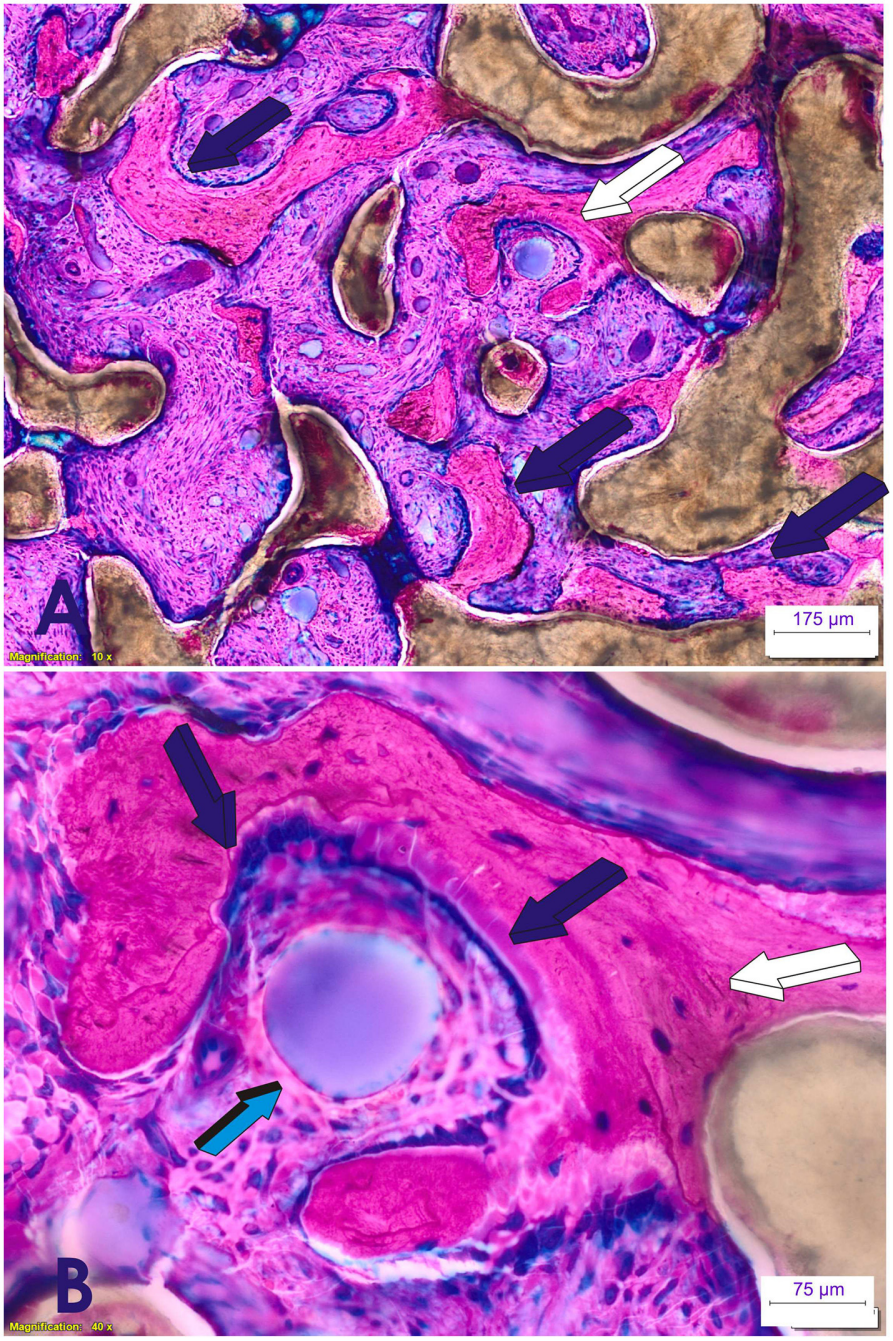
**Induction of bone formation by 125 μg recombinant human transforming growth factor-β_**3**_ (hTGF-β_**3**_)**. The recombinant morphogen was reconstituted with coral-derived macroporous bioreactors, implanted in the *rectus abdominis* muscle of *Papio ursinus*, and harvested on day 90 after heterotopic implantation (Ripamonti et al., 2015). **(A)** Florid induction of bone formation by the hTGF-β_3_ isoform with trabeculations of newly formed bone (dark blue arrows) across the macroporous spaces of the coral-derived bioreactor. (**B** high power view of **A**, white arrow) Plasticity of the newly formed bone molecularly cross-talking with the osteogenetic/morphogenetic central blood vessel (light blue arrow) that constructs the spatio/temporal plasticity of the newly formed bone covered by plumped osteoblastic-like cells (dark blue arrows) enveloping the invading morphogenetic central blood vessel. The plasticity of the two-dimension digital image shown in **(B)** is highlighted by the tractional bone movement from the coral-derived bioreactor which is supported by tractional collagenic fibers within the bone matrix (white arrow) that empower the newly formed bone to form around the central morphogenetic vessel enveloped by the plasticity movements of the newly formed bone as generated by the hTGF-β_3_. Thirty micrometer undecalcified sections prepared by using the Exakt cutting and grinding technique, stained with methylene blue basic fuchsin.

In the heterotopic bioassay for bone induction in rodents (Reddi, 2000), the three mammalian TGF-β isoforms do not initiate endochondral bone formation. Strikingly, the three mammalian isoforms, foremost the hTGF-β_3_ isoform, are powerful inducers of endochondral bone formation when implanted in the *rectus abdominis* muscle of the Chacma baboon *P. ursinus* when combined with either insoluble collagenous bone matrices (Figure 2; Ripamonti et al., 2008a) or coral-derived macroporous bioreactors (Figure 3; Klar et al., 2014; Ripamonti et al., 2014, 2015).

## The rapid induction of bone formaton in *Papio ursinus* by the recombinant human transforming growth factor-β_3_ isoform

In *Papio ursinus*, in marked contrast to rodents and lagomorphs, the hTGF-β_3_ protein induces large corticalized vascularized ossicles by day 30 after heterotopic implantation into the *rectus abdominis* muscle (Figure 2; Ripamonti et al., 2008a). Of note, substantial induction of bone formation is also achieved when the morphogen is combined with coral-derived macroporous bioreactors (Figure 3).

We undertook a series of experiments to examine the effects of 250 and 125 μg hTGF-β_3_ protein on osteoinduction. hTGF-β_3_ was added to coral-derived macroporous bioreactors and implanted into the *rectus abdominis* muscles of Chacma baboons. Implants were harvested 60, 30, and 15 days after implantation.

**Figure 4:**
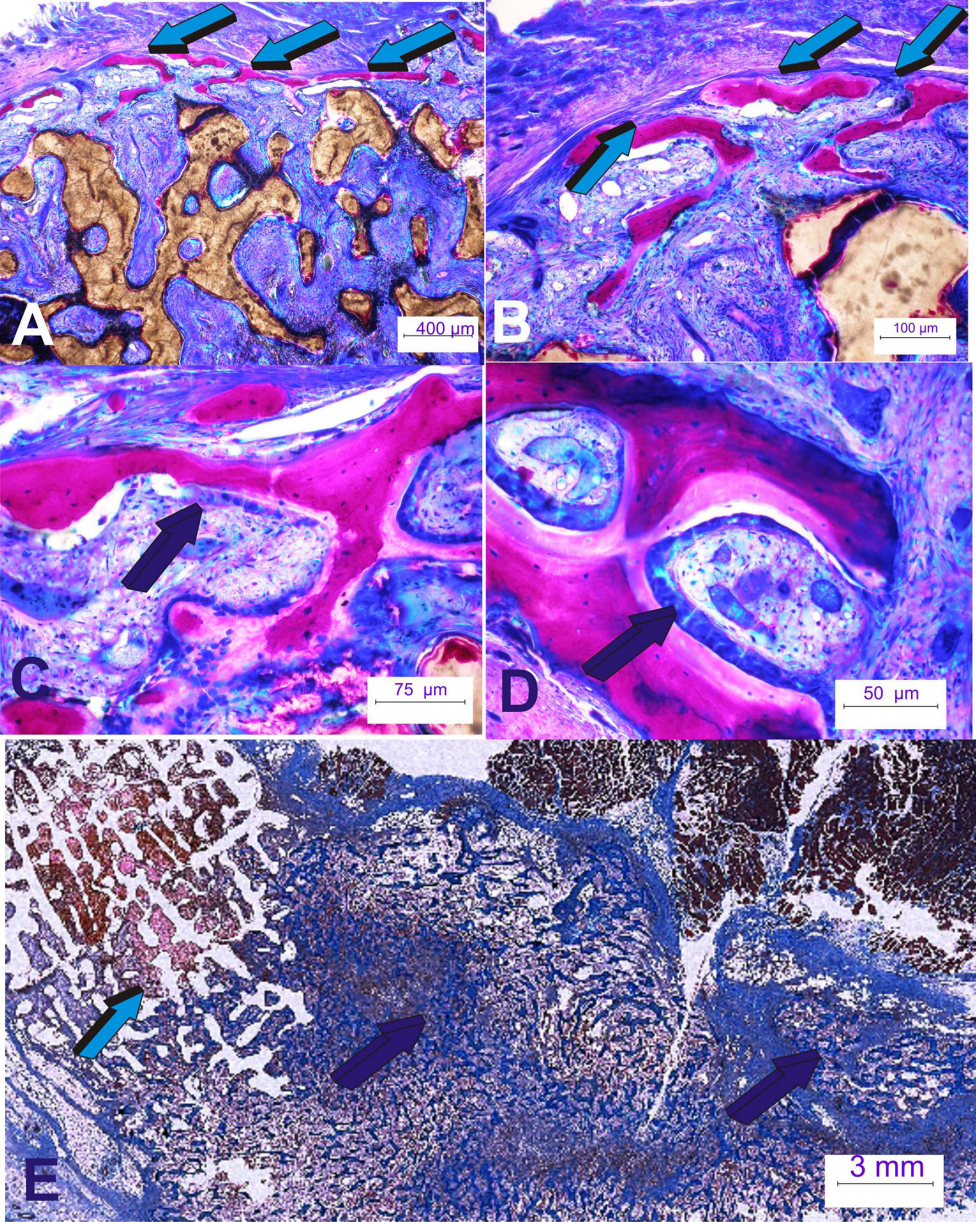
**Rapid spatio/temporally regulated tissue induction and morphogenesis by coral-derived macroporous bioreactors**. Bioreactors were pre-loaded with 250 μg recombinant human transforming growth factor-β_3_ (hTGF-β_3_). **(A,B)** Generated tissues were harvested on day 60 after intramuscular *rectus abdominis* implantation. Bone is initiated only at the very periphery of the implanted bioreactors (light blue arrows); there is lack of bone formation within the internal/central areas of the pre-loaded coral-derived constructs (Ripamonti et al., 2015). **(C,D)** High power views of the newly formed bone at the periphery of the macroporous constructs showing palisading of plumped osteoblastic-like cells (dark blue arrows) secreting osteoid matrix surfacing mineralized newly formed bone. **(E)** Massive induction of heterotopic bone formation by 250 μg doses hTGF-β_3_ extending few centimeters away from the preloaded coral-derived macroporous construct (light blue arrow) with lack of bone differentiation within the coral-derived macroporous bioreactor. Florid trabeculations of newly formed bone (dark blue arrows) extending several centimeters away from the coral-derived construct into the adjacent *rectus abdominis* muscle (dark blue arrows).

**Figure 5:**
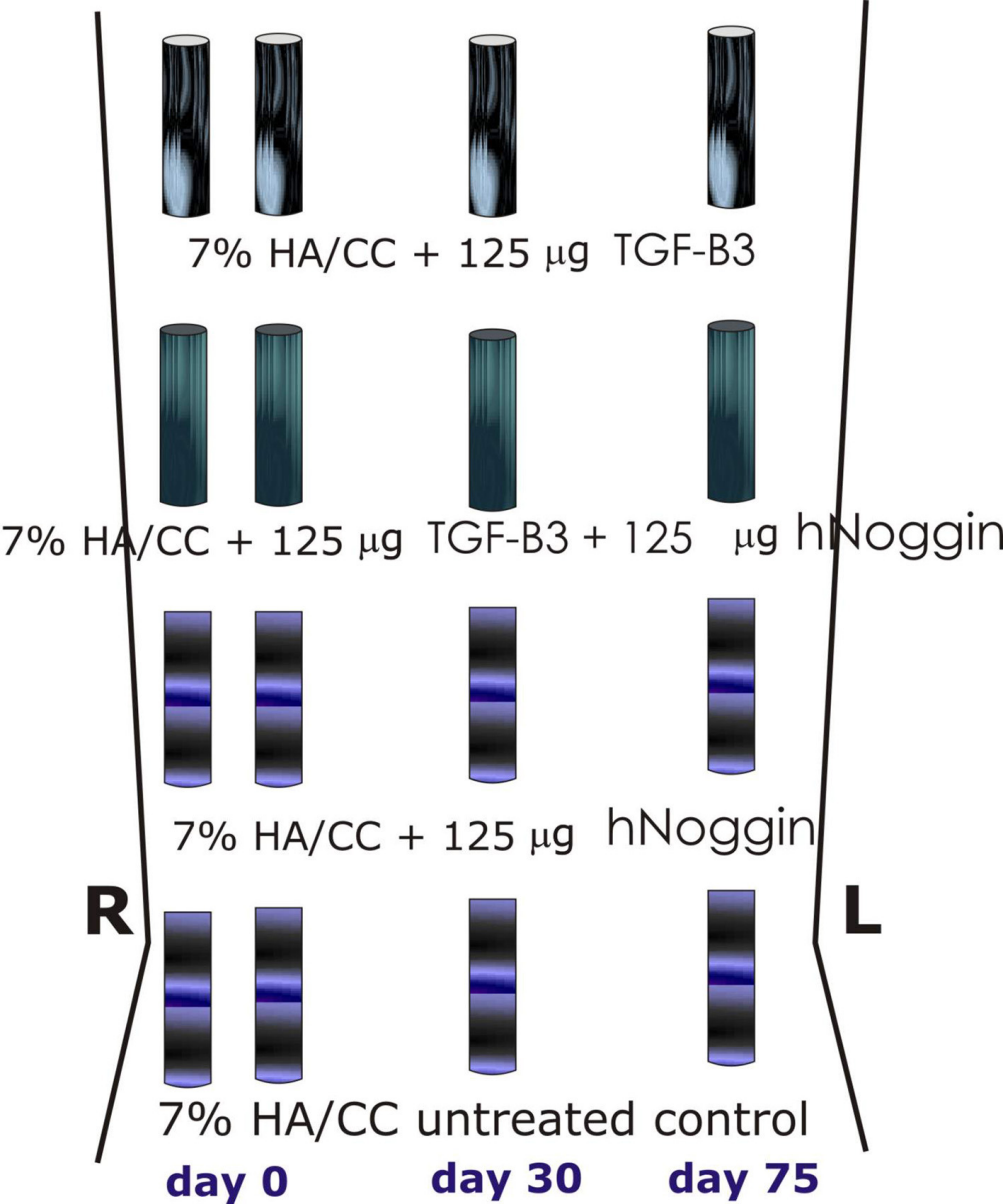
**Surgical model and implantation designs**. Heterotopic implantation protocols in the *rectus abdominis* muscle of the Chacma baboon *Papio ursinus* for tissue induction and morphogenesis by 7% hydroxyapatite/calcium carbonate coral-derived replicas preloaded with recombinant human transforming growth factor-β_3_ (hTGF-β_3_) with or without 125 or 150 μg recombinant human Noggin (hNoggin). Generated constructs were harvested on day 90, 60, and 15 after intramuscular heterotopic implantation and subjected to molecular and morphological analyses as described (Klar et al., 2014; Ripamonti et al., 2015).

The *rectus abdominis* striated muscle contains responding stem cells, distributed in different “*niches*” in perivascular/paravascular locations, that contribute a continuous flow of responding progenitor cells to initiate and enhance the bone induction cascade (Crisan et al., 2008; Ripamonti et al., 2008a, 2014; Ripamonti and Roden, 2010). Conclusively, in *P. ursinus* the hTGF-β_3_ protein initiated substantial induction of bone formation (Figures 2–4; Ripamonti et al., 2008a, 2014, 2015; Klar et al., 2014). Implantation schemes are shown in Figure 5 (Klar et al., 2013, 2014; Ripamonti et al., 2014, 2015).

When treating coral-derived macroporous bioreactors with 250 μg hTGF-β_3_ there is prominent induction of bone formation by day 60 at the very periphery of the implanted constructs, with limited induction of bone formation within the macroporous spaces of the heterotopically implanted coral-derived super-activated bioreactors (Figure 4; Klar et al., 2014; Ripamonti et al., 2014, 2015).

**Figure 6:**
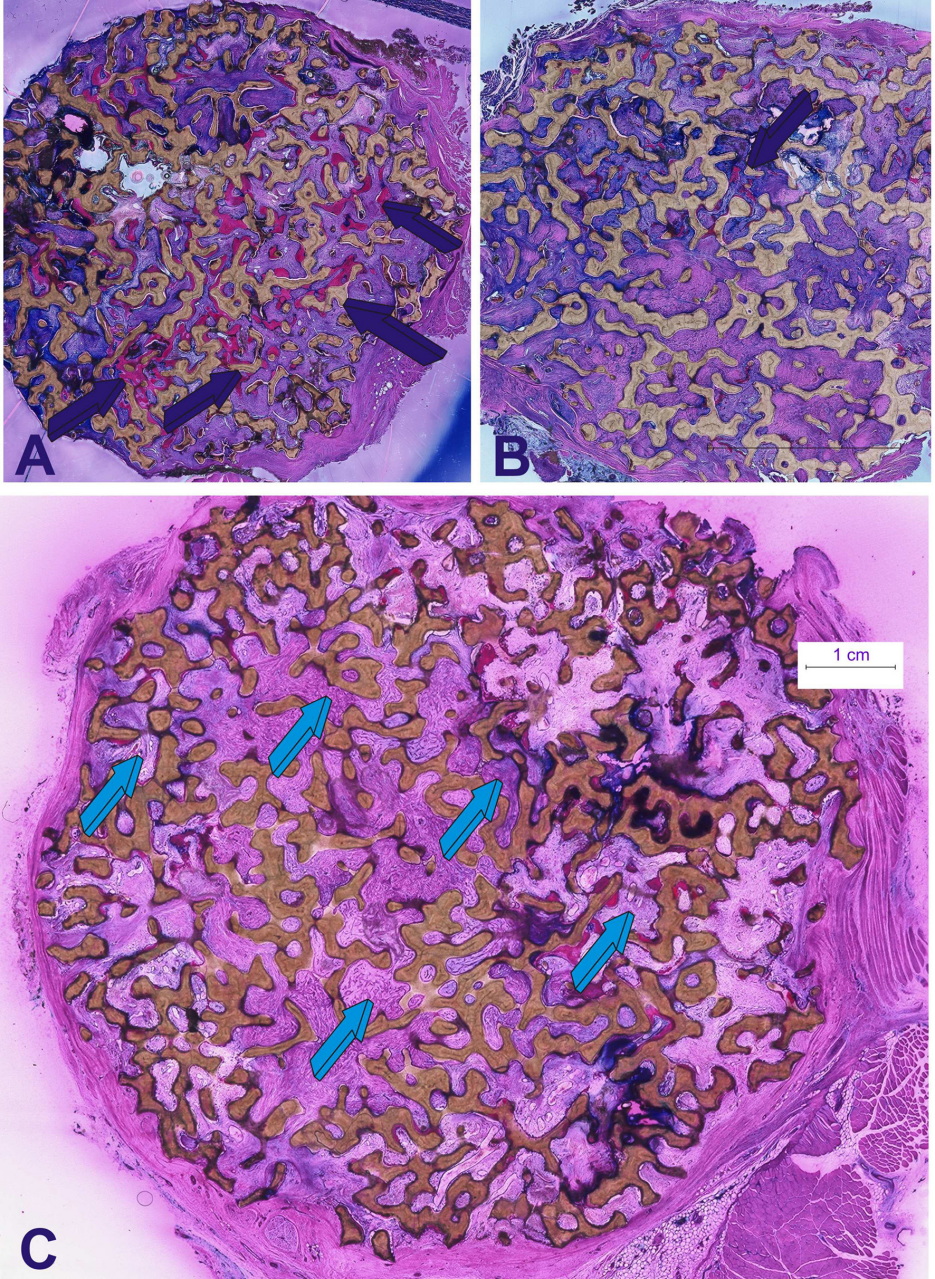
**Morphological data mechanistically defining the induction of bone formation by doses of the recombinant human transforming growth factor-β_**3**_**. hTGF-β_3_ was combined with coral-derived macroporous bioreactors and implanted in intramuscular *rectus abdominis* sites. Generated constructs were harvested on day 90 (Klar et al., 2014; Ripamonti et al., 2015). **(A)** Induction of bone formation (dark blue arrows) by 125 μg hTGF-β_3_ pre-loaded onto a macroporous coral-derived construct. What it is that set into motion the induction of bone formation by the hTGF-β_3_ isoform in primates? Is it still a bone morphogenetic proteins-related gene pathway found in primates only or, in primates, the highest phylogenetically evolved species, is it a different and altogether novel molecular pathway? Differences in gene regulation between primates during development and in response to morphogens may result in differences in sensitivity and responsiveness to TGF-β superfamily members (Ripamonti and Roden, 2010). Nature relies on common, yet limited molecular mechanisms, tailored to provide the emergence of specialized tissues and organs (Ripamonti et al., 1997). The bone morphogenetic proteins' family is indeed an elegant example of Nature's parsimony in programming multiple specialized functions deploying molecular isoforms with minor variations in amino acid motifs within highly conserved carboxy-terminal regions (Ripamonti, 2003, 2006). Macroporous bioreactors with or without the addition of 125 μg hTGF-β_3_ were additionally pre-loaded with 150 μg hNoggin, a known antagonist of BMPs signaling which inhibits the binding of selected BMPs to their receptors (for review see Ripamonti et al., 2014). **(B,C)** The addition of 150 μg hNoggin profoundly blocks the bone induction cascade by the pre-loaded hTGF-β_3_ protein. Morphological analyses on day 90 shows limited if any bone formation by induction (dark blue arrow in **B**) in macroporous bioreactors pre-treated with binary application hTGF-β_3_/hNoggin (light blue arrows in **C**). The use of 125 (Klar et al., 2014; Ripamonti et al., 2014) or 150 μg hNoggin (Ripamonti et al., 2015) in binary application with 125 μg hTGF-β_3_ has elegantly shown that the complex and apparently redundant pleiotropic activities of the osteogenic proteins of the TGF-β supergene family (Ripamonti, 2003; Ripamonti et al., 2014) are controlled by the mammalian TGF-β_3_ gene, and that the induction of bone formation, as initiated by the hTGF-β_3_ isoform when implanted in the *rectus abdominis* muscle of *Papio ursinus*, is via the *BMPs* pathway with hTGF-β_3_ controlling the induction of bone formation by regulating *BMPs* expression *via Noggin* expression (Ripamonti et al., 2014, 2015). Thirty micrometer undecalcified sections prepared by using the Exakt cutting and grinding technique, stained with methylene blue basic fuchsin.

We hypothesized that TGF-β signaling induces endochondral bone formation by regulating Noggin expression. Noggin is a known antagonist of BMPs signaling which inhibits the binding of selected BMPs to their receptors, thus blocking BMPs activities and resulting in a substantial decrease of bone formation (Ripamonti and Roden, 2010; Klar et al., 2014; Ripamonti et al., 2014, 2015). If the above listed cellular and molecular scenarios of activation and/or inhibition of BMP/TGF-β family members are correct, the addition of recombinant human Noggin in binary application with doses of the hTGF-β_3_ would inhibit the osteogenic activity of the expressed and secreted BMPs. We found that the addition of hNoggin to macroporous bioreactors pre-loaded with hTGF-β_3_ limited and/or blocked the bone induction cascade (Figure 6).

**Figure 7:**
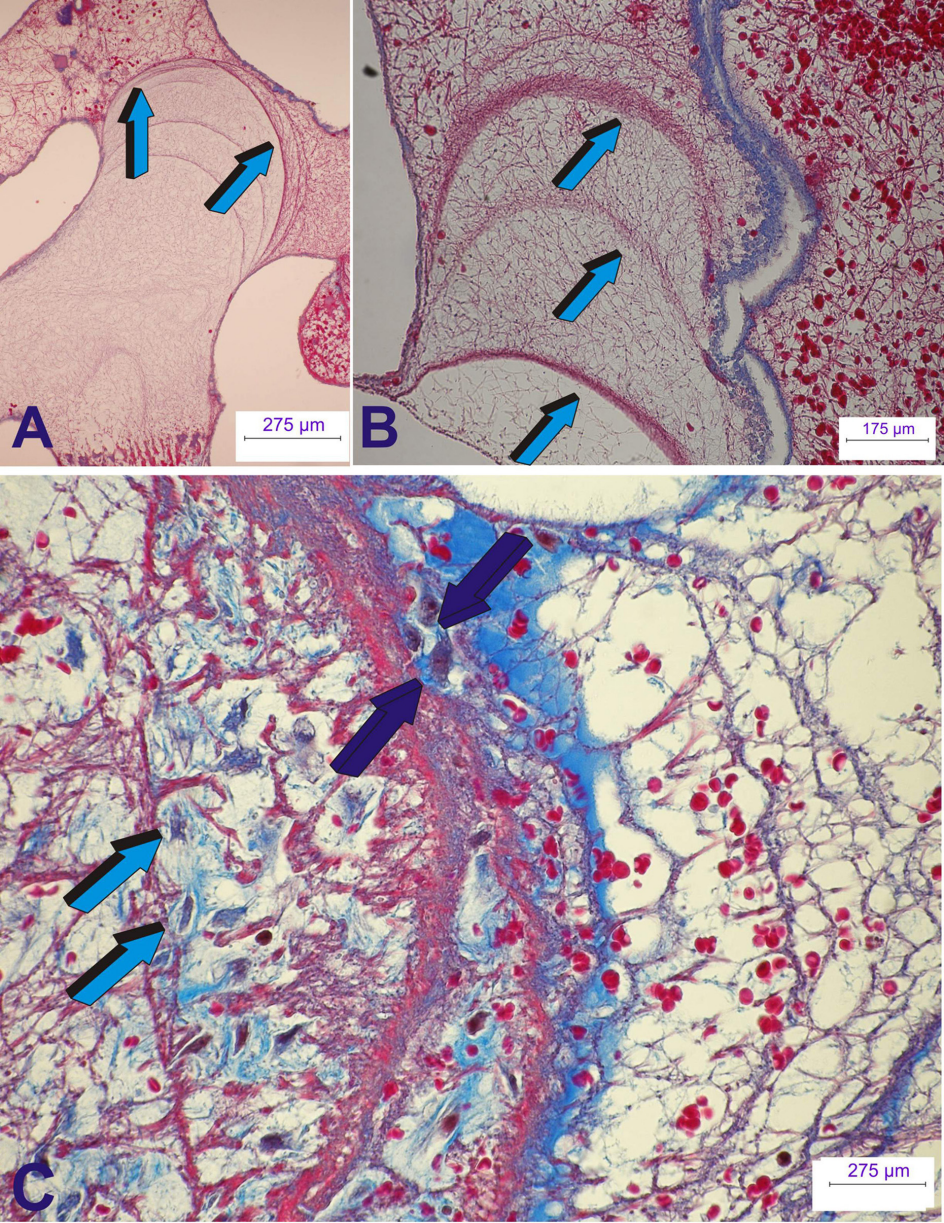
**Early tissue induction and morphogenesis by 125 recombinant human transforming growth factor-β_**3**_ (hTGF-β_**3**_)**. Doses of hTGF-β_3_ were pre-loaded into coral-derived macroporous bioreactors harvested on day 15 after intramuscular *rectus abdominis* implantation. **(A,B)** Extracellular matrix induction and tissue patterning assembling the complex induction of fibrin/fibronectin rings expanding within the invaded macroporous spaces (Klar et al., 2014; Ripamonti et al., 2014). **(A)** Differentiation and expansion of fibrin/fibronectin rings (light blue arrows) within the macroporous spaces. **(B)** Expanding rings (light blue arrows) move by compressing the extracellular matrix ahead of the driving ring. **(C)** Complex organized extracellular matrix rings provide structural anchorage to differentiating cells trapped and attached to the highly organized extracellular matrix (dark blue arrows). Aligned cells, interpreted as re-programmed pericytic/paravascular and/or myoblastic cells into pluripotent stem cells by the hTGF-β_3_ isoform are deployed for the rapid induction of newly differentiated osteoblastic cells nested and proliferating within the supportive extracellular matrix substratum (dark blue and light blue arrows). Thirty micrometer undecalcified sections prepared by using the Exakt cutting and grinding technique, stained with methylene blue basic fuchsin.

Molecularly, hTGF-β_3_/treated bioreactors significantly up-regulated the expression of *Runx2* and *Osteocalcin* (transcription factors associated with osteoblast differentiation). These control the differentiation of progenitor stem cells into the osteoblastic lineage (Klar et al., 2014; Ripamonti et al., 2014). Morphological analyses on day 15 (Figure 7) showed engineered microenvironments superactivated by hTGF-β_3_ reprogramming the recruitment of differentiated myoblastic and/or pericytic cells into highly active secreting osteoblasts in the *rectus abdominis* striated muscle of *P. ursinus*. The induction and expansion of fibrin-fibronectin extracellular matrix rings mechanistically predate the induction of a guided extracellular matrix microenvironment. This provides matrix cues for differentiating capillaries and sprouting angiogenesis for rapid cellular differentiation into osteoblastic-like cells secreting bone matrix, necessary for the induction of bone formation (Figure 7).

Up-regulation of *Runx2* and *Osteocalcin* on day 15 in hTGF-β_3_/treated macroporous bioreactors predates rapid cellular differentiation followed by substantial induction of bone formation by day 30 (Ripamonti et al., 2014). By contrast hNoggin/bioreactors down- regulate *BMP-2* expression. Down regulation of *BMP-2* (critical in bone development) correlated with minimal bone formation (Klar et al., 2014; Ripamonti et al., 2014, 2015). Binary applications of hTGF-β_3_-hNoggin/bioreactors profoundly inhibited *BMP-2* expression on day 15 and 60 but not on day 90 demonstrating a temporal control of bone differentiation (Klar et al., 2014; Ripamonti et al., 2014, 2015).

## Tissue patterning and morphogenesis by the transforming growth factor-β_3_

Our systematic experimentation in the non-human primate *P. ursinus* has confirmed our hypothesis that the induction of bone formation as initiated by doses of the hTGF-β_3_ isoform, is set into motion by the expression of a variety of *BMPs* genes. The profiled expressed *BMPs* result in the secretion of gene products that initiate the cascade of bone differentiation in the *rectus abdominis* muscle of *P. ursinus* (Ripamonti et al., 2014, 2015).

Critical for the mechanistic understanding of the cascade of bone formation by pre-treated and untreated macroporous bioreactors was the harvesting and molecular processing of the *rectus abdominis* muscle surrounding the implanted bioreactors (Ripamonti et al., 2015). Both the adjacent muscle and the bioreactor tissues were processed for molecular analyses which were correlated to the induction of bone formation on decalcified and undecalcified specimens (Ripamonti et al., 2015). The molecular analyses of the adjacent surrounding muscle tissues *vs*. the coral-derived bioreactor homogenates have helped to mechanistically resolve the pattern of the induction of bone formation initiated by the untreated vs. the hTGF-β_3_/treated macroporous constructs (Ripamonti et al., 2014, 2015). Furthermore, the molecular dissection of the adjacent *rectus abdominis* muscle tissue has generated additional knowledge on the rapid induction of bone formation at the periphery of the implanted super-activated bioreactors by the 250 μg doses of the hTGF-β_3_ (Ripamonti et al., 2015).

**Figure 8:**
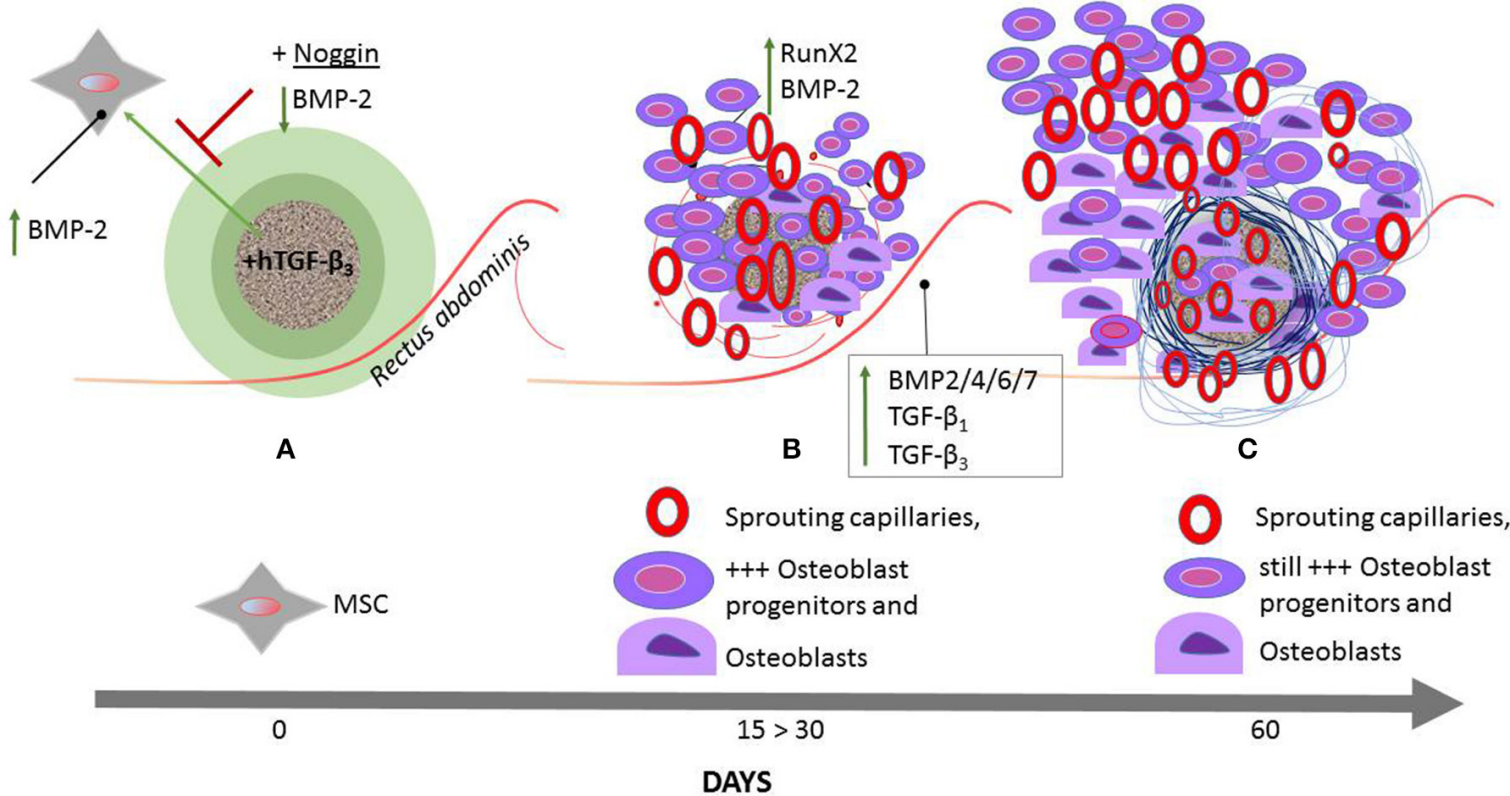
**Schematic representation of the induction of bone formation by the recombinant human transforming growth factor-β_**3**_ (hTGF-β_**3**_) combined with calcium carbonate coral-derived macroporous bioreactors**. hTGF-β_3_/treated coral derived macroporous bioreactors are implanted in the *rectus abdominis* muscles of the Chacma baboon *Papio ursinus* **(A)** and harvested on day 15, 30 **(B)**, and 60 **(C)**. The *rectus abdominis* muscle contains responding mesenchymal stem cells (MSC) **(A)** that set into motion the bone induction cascade. hTGF-β_3_/treated bioreactors significantly upregulate *Runx2* and *Osteocalcin* on day 15 **(B)**. The engineered microenvironment drives the recruitment of differentiated myoblastic and/or pericytic cells into active secreting osteoblasts **(B)**. The induction and expansion of fibrin/fibronectin extracellular matrix rings (Figure 7) within the macroporous spaces predates the induction of the guided extracellular micro-environment providing the cues for differentiating capillaries and angiogenesis **(B,C)**. hNoggin treated devices by contrast down- regulate *BMP-2* expression and correlates with minimal bone formation **(A)**. Between day 15 and 30 **(B)** there is rapid recruitment of osteoblast progenitors with differentiation of osteoblastic-like cells predominantly at the periphery of the implanted bioreactors with rapid induction of bone formation by day 30–60. Of great interest to note is that with the rapid induction of bone in treated devices **(B)**, there is the analogous expression of *BMP* and *TGF*-β*s* in the muscle tissue surrounding the macroporous treated devices. On day 60, in bioreactors treated with 250 μg hTGF-β_3_, there is a prominent induction of bone at the periphery of the implanted macroporous bioreactors with very limited bone formation within the macroporous spaces of the treated-bioreactors. The up- regulation of selected morphogenetic genes in the muscular and angiogenic tissues surrounding the implanted bioreactors is responsible for the rapid and substantial induction of bone formation by day 30 and 60 after implantation with newly formed bone well beyond the profile of the implanted macroporous constructs **(B,C)**. Terminal bars (T) represent repression; an upward green arrow indicates up- regulation.

Figure 8 schematically represents the *connubium* of all the morphological and molecular experimentation on the initiation of heterotopic bone formation by hTGF-β_3_/treated macroporous bioreactors implanted in the *rectus abdominis* muscle of *P. ursinus*.

Our studies of heterotopic *rectus abdominis* implantation have shown that the primary differentiating events that induce bone formation by untreated macroporous bioreactors develop within the macroporous spaces. This is associated with lack of, or minimal *BMP-2* gene expression within the surrounding adjacent muscle (Ripamonti et al., 2015). By contrast, in 250 μg hTGF-β_3_/treated bioreactors, both the adjacent muscle and the macroporous construct show *BMP-2* up-regulation (Figure 8), relating to the temporo/spatial rapid induction of bone formation at the periphery of the implanted bioreactors (Figures 4, 8). Of great interest to molecularly dissect the rapid induction of bone formation by the high doses of hTGF-β_3_ at the periphery of the pre-treated macroporous bioreactors, the adjacent muscle tissue shows the expression of several initiating and morphogenetic genes including but not limited to *BMP-3, BMP-4, BMP-6, BMP-7*, and *TGF*-β_1_, *TGF*-β_3_ and limited *TGF*-β_2_ expression (Figure 8; Ripamonti et al., 2015).

We conclude that a variety of profiled *BMPs* and *TGF*-β genes, that are expressed at different time points, temporally, and spatially regulate the induction of bone formation. These set into motion the bone induction cascade as initiated by the hTGF-β_3_ osteogenic device in the primate model (Ripamonti et al., 2015). In primates, the spatial and temporal expression of several profiled genes expressed after the implantation of the recombinant hTGF-β_3_ isoform controls the complex multicellular and multigene cascade of the induction of bone formation (Figure 8).

The data once again challenges the established paradigm of the induction of bone formation in primates (Ripamonti et al., 2014, 2015). The rapid and robust induction of bone formation is initiated by the hTGF-β_3_ isoform when reconstituted with either insoluble collagenous bone matrices or coral-derived macroporous bioreactors. This has shown that the hTGF-β_3_ isoform is a powerful soluble molecular signal that rapidly primes and induces available progenitor stem cells at a considerable distance from the implanted bioreactors from the surgically severed *rectus abdominis* muscle (Figures 4, 8).

## Tissue transfiguration *in vivo* by transforming growth factor-β supergene family members

**Figure 9:**
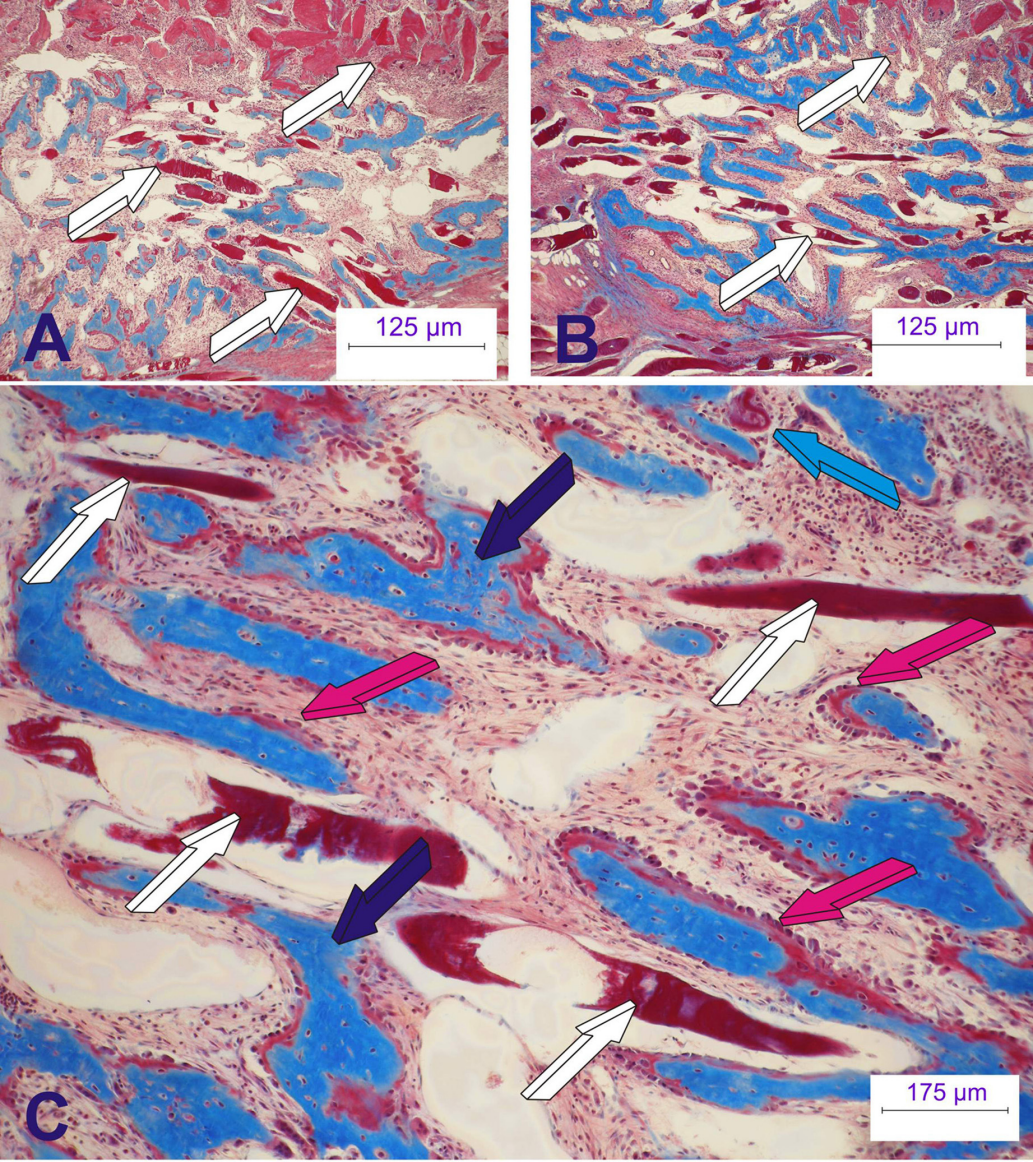
**The rapid biological activity of the recombinant human transforming growth factor-β_**3**_ (hTGF-β_**3**_)**. Morphological and molecular re-evaluations have indicated that the hTGF-β_3_ isoform “transfigures” mesenchymal tissues/muscle into bone. The synergistic induction of bone formation as engineered by binary application of recombinant human osteogenic protein-1 (hOP-1) with relatively low doses of hTGF-β_1_ at the optimal ratio 20:1 (hOP-1: hTGF-β_1_ or –β_3_) (Ripamonti et al., 1997, 2010) has recently introduced the molecular and morphological scenarios of the direct “tissue transfiguration” *in vivo*, a term that now defines the molecular and morphological evidence of the rapid induction of bone formation in primate tissue only, rapidly transfiguring the striated *rectus abdominis* muscle into bone. **(A,B)** Transfiguration of the *rectus abdominis* muscle (white arrows) into bone by the synergistic induction of bone formation by day 15 after heterotopic implantation. **(C)** High power view showing the rapid transfiguration of the striated muscle into bone with newly formed mineralized bone (dark blue arrows in **C**) surfaced by osteoid seams populated by contiguous osteoblasts (magenta arrows) with muscle fibers (white arrows) possibly degenerating after direct transformation into bone with osteoid formation (magenta arrows) facing the differentiation of haemopoietic cells and marrow development as early as 15 days after heterotopic implantation. Six micrometer undecalcified sections stained free-floating with modified Goldner's trichrome stain.

Of interest, the substantial and robust induction of bone formation by hTGF-β_3_ in the non-human primate *P. ursinus* has now forced a re-evaluation of the mechanistic insights of the induction of bone formation in primates, including humans (Ripamonti et al., 2014, 2015). The hTGF-β_3_ isoform does not merely initiate the induction of bone formation but also sets the molecular and morphological rules of the direct “*tissue transfiguration in vivo*” (Ripamonti, 2012, 2015a,b). This term defines the molecular and morphological evidence of the induction of bone formation in primate tissues rapidly transfiguring the striated *rectus abdominis* muscle into bone *in vivo* (Figure 9).

The morphological and molecular evidence of the rapid transfiguration of muscle tissue into bone by hTGF-β_3_ (Ripamonti et al., 2008a, 2014, 2015; Klar et al., 2014) is further potentiated by the synergistic interaction with osteogenic protein-1 (hOP-1) (Ripamonti et al., 1997; Figure 9). This has indicated to the Bone Research Laboratory a novel and unexplored biological function of the hTGF-β_3_ isoform (Ripamonti et al., 2016). Injections of hTGF-β_3_ into malignant neoplastic primary and secondary masses may induce the rapid transfiguration of the injected masses into bone to facilitate tumoral ablation and its surgical debridement (Ripamonti, 2012, 2015a,b). We hypothesize that hTGF-β_3_ would “*osteogenize*” the tumor, transfiguring all available responding cells into osteoblastic-like cells, possibly altering not only the neoplastic phenotype but also the neoplastic genotype (Ripamonti, 2012, 2015b), thus controlling differentiation so as to osteogenize secondary masses.

Lastly, our research does not as yet offer molecular insights into why the induction of bone formation by the mammalian hTGF-β proteins occurs in primates only. In previous work, we have suggested that the above research question should be a mandatory research goal so as to resolve the induction of bone formation by the mammalian hTGF-β proteins in primates (Klar et al., 2014; Ripamonti et al., 2014). We have proposed the presence of selective molecular redundancy signals amongst the members of the TGF-β supergene family with a multifactorial tuning of speciation-related molecular evolution in anthropoid apes at the early Pleistocene boundary (Klar et al., 2014), that resulted in a tighter control of the bone induction process in primates species.

## Author contributions

UR, Research planning, animal experimentation, evaluation of data, write-up. RD, Molecular biology planning and execution, planning of results. RP, Research planning, specialized histo-technological techniques, staining and histomorphometric analysis. CD, Planning of molecular analyses, interpretation of result data, write-up. TD, Molecular analyses, planning and execution, evaluation of results. RK, Molecular analyses, planning and execution, submission online.

## Funding

Funding for continuous research on the osteoinduction prerogative of the recombinant hTGF-B inform were from the SA National Research Foundation, the University of the Witwatersrand, Johannesburg, and from *ad-hoc* grants of the Bone Research Laboratory.

### Conflict of interest statement

The authors declare that the research was conducted in the absence of any commercial or financial relationships that could be construed as a potential conflict of interest. The reviewer PP and handling Editor declared their shared affiliation, and the handling Editor states that the process nevertheless met the standards of a fair and objective review.
